# Study on exosomes for identifying bipolar disorder in early stage: A cross‐sectional and validation study protocol

**DOI:** 10.1002/brb3.3494

**Published:** 2024-04-19

**Authors:** Yanqing Wu, Yuchao Li, Xuguang An, Jiangong Li, Chenghao Yang, Yi Wang

**Affiliations:** ^1^ Tianjin Mental Health Center Tianjin Anding Hospital Tianjin China

**Keywords:** bipolar disorder, early diagnosis, exosome metabolite, major depression

## Abstract

**Background:**

The difficulty is remained to accurately distinguish bipolar disorder (BD) from major depressive disorder (MDD) in early stage, with a delayed diagnosis for 5–10 years. BD patients are often treated with antidepressants systematically due to being diagnosed with MDD, affecting the disease course and clinical outcomes. The current study aims to explore the role of plasma exosomes as biomarker to distinguish BD from MDD in early stage.

**Methods:**

Two stages are included. The first stage is a cross‐sectional study, comparing the concentrations of plasma exosome microRNA and related proteins among BD group, MDD group, and healthy controls (HC) group (*n* = 40 respectively), to identify target biomarkers preliminarily. The “Latent Class Analysis” and “Receiver Operating Characteristic” analysis will be performed to determine the optimal concentration range for each biomarker. The second stage is to validate target markers in subjects, coming from an ongoing study focusing on patients with a first depressive episode. All target biomarkers will be test in plasma samples reserved at the initial stage to detect whether the diagnosis indicated by biomarker level is consistent with the diagnosis by DSM‐5. Furthermore, the correlation between specific biomarkers and the manic episode, suicidal ideation, and adverse reactions will also be observed.

**Discussion:**

Exosome‐derived microRNA and related proteins have potential in serving as a good medium for exploring mental disorders because it can pass through the blood‐brain barrier bidirectionally and convey a large amount of information stably. Improving the early diagnosis of BD would help implement appropriate intervention strategy as early as possible and significantly reduce the burden of disease.

## 1 BACKGROUND

Bipolar disorder (BD) is a chronic and recurrent mental illness, which affects over 1% of the global population and involves depressive and manic and/or hypomanic episodes, leading to severe cognitive impairments and disability of social functioning (Alonso et al., 2011). Regrettably, accurately distinguishing between BD and major depressive disorder (MDD) at the early stages is challenging in clinical practice, resulting in misdiagnosis and improper treatment. Only 20% of BD patients initiated with a depressive episode receive a correct diagnosis within the first year of onset (Hirschfeld et al., 2003), with an average delay of 5–10 years before an appropriate diagnosis (Baldessarini et al., 2007). The main causes of this dilemma include first, approximately 50% of BD patients initially exhibit depressive symptoms without clear signs of manic or hypomanic symptoms, rendering clinical manifestations strikingly similar to MDD (Drancourt et al., 2013; Tondo et al., 2010); second, BD typically emerges during adolescence and early adulthood (Bolton et al., 2021; Kelberman et al., 2021), a period when affected individuals tend to neglect abnormal emotional states, especially hypomanic symptoms, resulting in a low visiting rate (Post & Grunze, 2021); furthermore, the blurry boundary between hypomanic affect and normal emotions complicates clinical diagnosis (Bolton et al., 2021). Misdiagnosis often leads to inappropriate treatment, as BD patients misdiagnosed with MDD receive systematic treatments with antidepressants, affecting disease progression and clinical prognosis (Berkol et al., 2019; Post et al., 2010). Research indicates that antidepressant treatment can induce manic episodes or mixed states (Tondo et al., 2010), exacerbate depressive mood (Berkol et al., 2019), increase agitated behaviors (Tondo et al., 2010), and elevate suicide risk in bipolar‐depressed patients (Berkol et al., 2019). Simultaneously, our study suggests that early antidepressant treatment in BD patients can worsen cognitive impairment even during remission, including executive function, attention, and processing speed (unpublished, clinicaltrials.gov ID: NCT04564573).

Given the challenges in early diagnosis of BD and potential adverse effects, identifying reliable and valid biological markers for early‐stage BD diagnosis is of great clinical value. The unknown etiology of BD severely hampers the study of specific biological markers. Although various pathological hypotheses have been proposed, such as the monoamine hypothesis, glutamate hypothesis, glial cell dysfunction, and neuroinflammation hypothesis, none can fully clarify its pathogenesis or explain clinical symptoms (Sigitova et al., 2017). In recent years, neuroimaging studies have gained attention in hopes of identifying characteristic changes in brain structure, functional status, neural transmission connections, and biochemistry in patients with mood disorder. However, due to sample heterogeneity and limitation of imaging technology, research outcome remains suboptimal in consistency (Kelberman et al., 2021). Likewise, numerous genomic studies have been conducted due to BD's high heritability; however, pathogenic genes remain unidentified (Hara et al., 2022), and research conclusions on gene polymorphism‐related risk variants are highly inconsistent and far from clinical application (Huang et al., 2020; Shinozaki & Potash, 2014). Additionally, although some serum markers expressed markedly differently between BD and MDD patients, these differences cannot effectively distinguish between the two conditions (Capuzzi et al., 2022; Rhee et al., 2020; Shahyad et al., 2023).

Exosomes are membrane‐bound vesicles, 40–160 nm in diameter, secreted by living cells into the extracellular matrix and found in various body fluids like blood. They contain diverse substances such as proteins and microRNA and serve multiple functions including antigen presentation, immune escape, cell transformation induction, among others (Yang et al., 2019). Furthermore, neuronal cell‐derived exosomes can cross the blood‐brain barrier, with the vesicle structure protecting their contents from degradation and damage during long‐distance transport (Fan et al., 2022; Terstappen et al., 2021). This feature enables us to understand biological activity of central nervous cell, functional status, and intercellular information transmission through peripheral blood (Fan et al., 2022). In this regard, exosomes would be a crucial medium for investigating psychiatric disorder pathogenesis by bridging peripheral and central systems.

Currently, exosome research in the psychiatry field is in its infancy, with few relevant literature reports, mainly focusing on exosomes as biological markers of disease status. Du et al. (2022) discovered that serum exosome metabolite dysregulation is related to BD onset and progression; Delalle (2021) found that the level of exosome miR‐149 is significantly increased in BD patients' anterior cingulate gyrus. Dysfunction of lncRNAs involved in inflammation and neurogenesis has been implicated in the pathogenesis of BD (Bella & Campo, 2021). Given the close association between exosomes and BD, we plan to explore exosomes as biomarkers for the early diagnosis of BD. In order to optimize the experimental design, we will (1) require a minimum of 5 years of medical history for patients in MDD group to reduce the risk of recruiting potential patients with BD; (2) include a healthy control (HC) group to minimize bias risk, as some previous studies only compare BD and MDD participants; (3) conduct a validation study to verify the diagnostic and predictive efficacy of the biomarkers, as previous studies have been limited to examining the statistical significance of markers in expression, which restricts expanding study conclusions to the clinical practice.

## 2 METHODS/DESIGN

### 2.1 Study design

This is a two‐stage trial with a cross‐sectional study and a clinical validation study. For the first stage, levels of plasma exosome microRNA and proteins will be test in BD, MDD and HC groups to explore target markers, which show significant differences and/or specific expression patterns in individuals with BD compared to MDD and HC groups. A clinical validation study will be conducted in the second stage and will recruit participants from an ongoing trial in which patients with first‐episode depression were followed up for 5 years at least. All participants will be divided into two groups (MDD and BD groups, *n* = 17 respectively) based on whether they have/had hypomanic or manic episodes currently or previously, according to the DSM‐5 diagnosed with SCID‐5. All target biomarkers will be test in plasma samples reserved at the initial stage to detect whether the diagnosis indicated by the biomarkers is consistent with diagnosis by DSM‐5. In addition to the accuracy of predicting diagnosis, the correlation between specific biomarkers and the manic episode (differentiating between bipolar I and II disorder), suicidal ideation, and adverse reactions will also be observed.

### 2.2 Setting

All procedures will be performed in Tianjin Anding Hospital, in addition to exosome analysis, such as detection and validation of exosome microRNA and related proteins.

### 2.3 Population

This study will consist of a sample of 154 participants (BD1 group vs. MDD1 group vs. HC group for the first stage: *n* = 40, respectively; BD2 group vs. MDD2 group for the second stage: *n* = 17, respectively) recruited from outpatient and inpatient departments of Tianjin Anding Hospital.

### 2.4 Inclusion and exclusion criteria

#### 2.4.1 Inclusion criteria

BD1/MDD1 group: (1) meets the diagnostic criteria for BD or MDD according to the DSM‐5 diagnosed with SCID‐5; (2) Han ethnicity, any gender, aged 18–60 years old; (3) initial onset were depressive episodes for BD patients; (4) a history of depression of no less than 5 years for MDD patients; (5) participants comply with all procedures of study; (6) participants must sign the informed consent.

HC group: (1) no mental disorders that meet the diagnostic criteria in DSM‐5; (2) Han ethnicity, any gender, aged 18–60 years old; (3) participants comply with all procedures of study; (4) participants must sign the informed consent.

#### 2.4.2 Exclusion criteria

(1) Meets the diagnostic criteria for other mental disorders in DSM‐5 for BD1/MDD1 groups; two generations of relatives have mental disorders that meet the diagnostic criteria in DSM‐5 for HC group; (2) accompanied by severe physical illnesses, including uncontrolled hypertension, severe cardiovascular, cerebrovascular, pulmonary diseases, thyroid diseases, diabetes, epilepsy, metabolic syndrome, etc.; (3) history of alcohol abuse or use of other psychoactive substances; (4) pregnant or lactating women; (5) any factors that hinder the participant from providing informed consent or participating in the study.

#### 2.4.3 BD2 and MDD2 groups

Participants come from an ongoing study. The drug‐naïve patients with the first episode of depression were recruited from outpatient department of Tianjin Anding Hospital, aged 18 to 50 years, both genders, diagnosed with DSM‐5. See the published article for details of inclusion and exclusion criteria (Wang et al., 2021).

### 2.5 Sample size calculation

Currently, there is limited research examining the metabolomic identification of exosomes‐derived biomarkers in BD. Existing research suggests that sample sizes of less than 40 individuals per group can yield significant results (Du et al., 2022). Therefore, our first stage will recruit 40 individuals per group. No consideration of loss to follow up for a cross‐sectional study.

The primary outcome of our study is to determine the accuracy of target biomarkers in differentiating BD from MDD through the calculation of the area under the curve (AUC) from receiver operating characteristic (ROC) analysis. Based on previous research, the AUC values for distinguishing BD from HC exceeded 0.80 (Du et al., 2022). To achieve a statistical power of 80% (1 – *β*) with an *α* level of 0.05, sample size calculations conducted using PASS 15 software with a two‐sided *t*‐test suggest that 17 individuals per group are required for the second stage.

### 2.6 Study endpoint

#### 2.6.1 Main study endpoints

The accuracy of predicting diagnosis, decided by the quotient: (1) the number of BD patients indicated by exosome‐derived markers**/**17 for BD2 group; and (2) 100% minus the number of BD patients indicated by exosome‐derived markers**/**17 for MDD2 group.

#### 2.6.2 Secondary study endpoints

The correlation between specific biomarkers and manic episode (Young Mania Rating Scale, YMRS), suicidal ideation (the Beck Scale for Suicide Ideation‐Chinese Version, BSS), and adverse reactions (Treatment Emergent Symptoms Scale, TESS) will also be inspected.

### 2.7 Duration

For the first stage study, the information collection and blood sampling for each participant will be finished within 30 min after the participant qualified for inclusion. It is anticipated to finish the inclusion work within 1 year. For the second stage, the ongoing study had started at the end of 2019 and has recruited 200 participants at least; all samples are ready and participants are followed up.

### 2.8 Additional study parameters

The information on age, sex, nation, family history of psychiatric disorder, age of onset, time of BD diagnosis, education level, number of depressive and manic episodes, and history of euthymia (yes/no) will be collected.

### 2.9 Blinding and study‐group allocation

The entire procedure does not involve blinding, and the subject allocation is based on the psychiatric state according to DSM‐5.

### 2.10 Study procedures

#### 2.10.1 Overview of procedure

**FIGURE 1:**
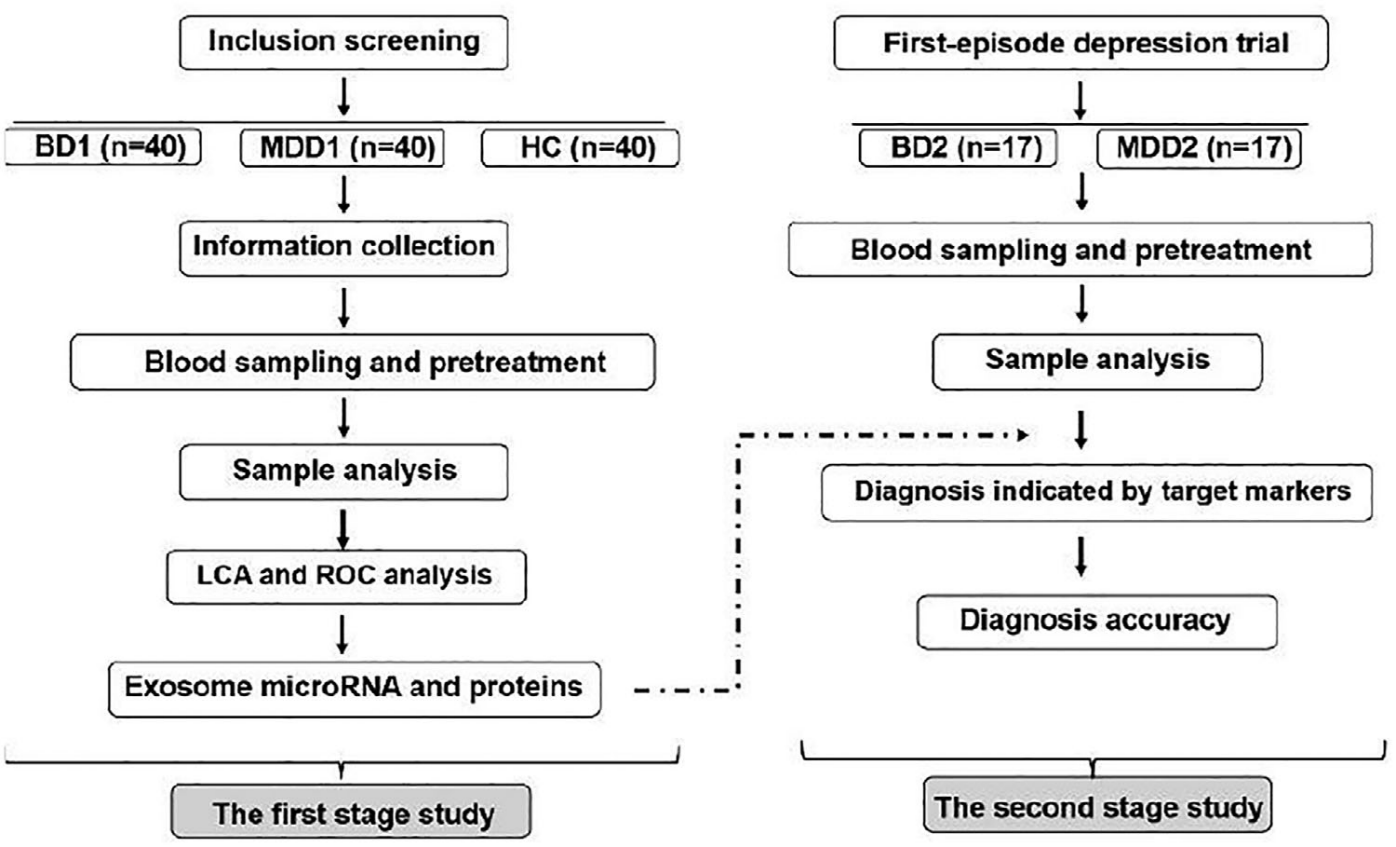
Study flowchart. BD, bipolar affective disorder; MDD, major depressive disorder; HC, healthy control; LCA, latent class analysis; ROC, receiver operating characteristic.

In the cross‐sectional study, the screening for inclusion will be finished by two psychiatrists separately. If the both reach a consensus on the condition, the individual can be included; otherwise, a higher‐ranking psychiatry will be consulted to review the case; and then the blood sampling and information collection will be done. The blood samples will be processed and then stored in a −80°C freezer for subsequent exosome analysis, which is took charge of by the biotech company. Comparing the levels of plasma exosome microRNA and related proteins among three groups (BD1, MDD1, and HC groups) to preliminarily identify target microRNA and proteins, which are significant different in BD1 group from MDD1 and HC groups. The LCA on target microRNA and proteins will be conducted on all samples to observe whether they can effectively distinguish BD patients from MDD patients and healthy participants. Then, based on the results of LCA, the ROC analysis will be performed to further determine the optimal concentration (cut‐off value) for each indicator and ultimately determine the target biomarkers. The second stage is a clinical validation study in which subjects, who come from an ongoing trial and initiated with a depressive episode and were followed up for 5 years at least, are divided into two groups (MDD2 and BD2 groups, *n* = 17 respectively) based on whether they have/had hypomanic and/or manic episodes currently or previously, according to the DSM‐5 diagnosed with SCID‐5. All target biomarkers will be test in plasma samples reserved at the baseline to detect whether the diagnosis indicated by biomarkers is consistent with diagnosis by DSM‐5 (the number of BD indicated by target markers in BD2 group and MDD2 group, respectively). As well as the accuracy of predicting diagnosis, the correlation between specific biomarkers and manic episode (bipolar I/II disorder), suicidal ideation, and adverse reactions will also be observed (see Figure 1 for study flowchart).

### 2.11 Scale assessments

BSS: to evaluate the suicidal ideation and suicidal risk during the illness course. To investigate the potential relationship between specific biomarkers and suicidal ideation, and to provide early warning signs of suicidal behavior by testing for these biomarkers at the onset of illness.

YMRS: to assess the severity of manic symptoms. It includes 11 items, with a score not less than 6 indicating a manic state. Among the patients with BD, it is expected to pick up the patients who may experience manic episodes but not hypomanic episodes only (refer to bipolar I disorder) in advance, then the use of antidepressants and mood stabilizers can be more guided.

TESS: to assess the adverse reactions that patients may experience during drug treatment. It includes multiple items that assess various adverse reactions, and each item has specific scoring criteria, with scores ranging from 0 to 4.

### 2.12 Biomarkers

#### 2.12.1 Plasma exosomes

Plasma samples are utilized to investigate the potential exosome‐derived microRNAs and proteins for the early diagnosis of BD. Ten milliliters of venous blood are adopted from each participant into an EDTA‐2k anticoagulant vacuum tube. The blood will be immediately centrifuged at 1500 × *g* for 15 min at room temperature, and plasma is kept frozen at −80°C until analysis.

### 2.13 Data management

Data will be entered into OpenClinica online with range check. The academic board of Tianjin Anding Hospital will be in charge of overseeing the trial including data monitoring, endpoint adjudication, and project implementation, which will be independent from investigators and the sponsor.

### 2.14 Medication use

None.

### 2.15 Side effects

None (the side effects emerged in the second stage are managed by the protocol its own, independent from the current study).

### 2.16 Safety reporting

#### 2.16.1 Adverse events (AE)/serious adverse events (SAE)

The first stage is a cross‐sectional study without any interventions. So, there is no need for AE/SAE monitoring based on the design of the study.

### 2.17 Withdrawal

#### 2.17.1 Withdrawal of subjects

Subjects can quit the study at any time for any considerations without any consequences. The investigator can decide to withdraw subjects from the trial if the subjects are not compliant to the study procedures or any conditions emerge which violating to inclusion/exclusion criteria during processing. All withdrawals will be excluded from the study and new participants will be recruited for replacing.

### 2.18 Statistical analysis

The data obtained from questionnaires and basic information are expressed in “mean ± standard deviation” in the case of normal distributions and in median and quartile in the case of nonnormal distributions. The chi square and analysis of variance (ANOVA) test will be used to test the consistency of match between groups for factors like age, sex etc., and the factors with significantly different distributions among groups will be used as covariates. The comparisons in scores of each scale and concentrations of exosome‐derived markers among three groups will be made by ANOVA. Additionally, the LCA and ROC analyses will be conducted by the SPSS 22.0 statistical analysis software to assess the potential of exosome microRNA and related proteins as biomarkers to distinguish BD patients from MDD patients and healthy participants. The test level is *α* = 0.05.

## 3 DISCUSSION

The early diagnosis of BD poses a significant challenge in clinical practice due to the absence of specific biomarkers and the complexities in distinguishing BD from MDD. Delayed diagnosis or misdiagnosis would lead to serious consequences, such as unsuitable medication, prolonged illness course, heightened risk of suicide, and diminished quality of life for patients. Research endeavors have targeted potential methods for the early diagnosis of BD. Some investigations have employed neuroimaging techniques to examine structural and functional brain anomalies in BD patients during manic and interepisode phases and shown positive outcomes (Claeys et al., 2022; Siegel‐Ramsay et al., 2022; Sunaga et al., 2020). Other research has explored differences in peripheral blood inflammatory cytokines and paraoxonase1 Q192R gene polymorphism between patients with BD and MDD (Bortolasci et al., 2014). Recently, a predictive model that incorporates oxidative stress factors has also demonstrated potential in diagnosing BD (Niu et al., 2022). Nonetheless, the clinical applicability of these approaches is limited by various factors, such as small sample sizes, heterogeneous study populations, and the restricted reliability and validity of biomarkers. As a result, future research in this area should be focused on targeted improvements aimed at enhancing research efficiency. For instance, enhancing the early diagnosis of BD necessitates larger sample sizes, increased homogeneity of participants, the development of more cost‐effective diagnostic techniques, and more reliable biomarkers. Moreover, validation studies should be undertaken to confirm the validity and reliability of potential diagnostic biomarkers. Exosomes have recently gained attention as a possible biomarker for the early diagnosis of BD as shown previously, owing to their noninvasive extraction from body fluids, the sensitivity to different states of brain (Fan et al., 2022), and the capacity to transport various biological molecules (Fan et al., 2022; Terstappen et al., 2021). Additionally, exosomes can traverse the blood‐brain barrier freely (Terstappen et al., 2021), facilitating a better comprehension of the central nervous system's functional status. In this regard, exosomes can offer a more comprehensive insight into the biological characteristics and pathophysiological basis of BD.

Considering the shortages of the previous studies, we plan to make specific and deliberate adjustments to increase the power of the current study. First, there is a high risk of recruiting participants who would develop into patients with BD, so it requires a minimum of 5 years of medical history for MDD patients. Second, it will include a MDD group to minimize bias risk because some preceding studies only made comparison between BD group and HC group. Third, we will conduct LCA and ROC analyses in order to identify a more precise and effective range of biomarker concentrations, in terms of improving the operability of research outcome and clinical generalizability. Most importantly, we will conduct a validation study to verify the diagnostic and predictive efficacy of the target biomarkers, as previous studies have been limited to examining the statistical significance of biomarkers in expression.

## 4 LIMITATIONS

There are some limitations of the current study. First, a 5‐year history of MDD cannot completely rule out the possibility of recruiting potential BD patients in MDD group. Second, the sample size is relatively small for an exosome study. Finally, we do not make a discrimination between BD I type and BD II type in BD group, the different underlying pathophysiological mechanism between two conditions may mediate the expression of exosome microRNA and related proteins.

## 5 CONCLUSION

In summary, despite these limitations, the current study is still expected to help us look further into the efficacy of exosome microRNA and related proteins in early diagnosis of BD and may provide clues for inspecting the pathophysiological association/discrepancy between MDD and BD.

## AUTHOR CONTRIBUTIONS

Chenghao Yang and Yi Wang conceived the study. Yanqing Wu and Yuchao Li drafted the first version of manuscript. Xuguang An and Jiangong Li provided the methodological supports and reviewed the related section. Chenghao Yang acquired funding. Chenghao Yang and Yi Wang finished the review and editing of protocol. All authors provided input and read and approved the final version.

## CONFLICT OF INTEREST STATEMENT

The authors declare that they have no known competing financial interests or personal relationships that could have appeared to influence the work reported in this paper.

## ROLE OF THE FUNDING SOURCE

The funding sponsor is not involved in the study design, the collection, the analysis and interpretation of data, the writing of the report, and the decision for publication.

## PATIENT CONSENT STATEMENT FOR WORK WITH HUMAN SUBJECTS

All participants must sign informed consent of the study prior to the enrollment.

## TRIAL REGISTRATION

The study was registered on Clinicaltrials.gov with protocol ID (2022KJ264) and clinicaltrials.gov ID (NCT05915312).

### PEER REVIEW

The peer review history for this article is available at https://publons.com/publon/10.1002/brb3.3494.

## Data Availability

The related data and materials are available from the corresponding author on reasonable request.
